# Interrelations of aortic spring function, cardiovascular disease risk factors, and left ventricular diastolic function: The Framingham Heart Study

**DOI:** 10.14814/phy2.70985

**Published:** 2026-06-30

**Authors:** Leroy L. Cooper, Brenton R. Prescott, Vanessa Xanthakis, Jian Rong, Martin G. Larson, Emelia J. Benjamin, Naomi M. Hamburg, Ramachandran S. Vasan, Gary F. Mitchell

**Affiliations:** ^1^ Biology Department Vassar College Poughkeepsie New York USA; ^2^ Boston University and NHLBI's Framingham Study Framingham Massachusetts USA; ^3^ Section of Preventive Medicine and Epidemiology, Department of Medicine Boston University Chobanian and Avedisian School of Medicine Boston Massachusetts USA; ^4^ Department of Biostatistics Boston University School of Public Health Boston Massachusetts USA; ^5^ Section of Cardiovascular Medicine, Department of Medicine Boston University Chobanian & Avedisian School of Medicine Boston Massachusetts USA; ^6^ Department of Epidemiology Boston University School of Public Health Boston Massachusetts USA; ^7^ Evans Department of Medicine & Whitaker Cardiovascular Institute Boston University Chobanian & Avedisian School of Medicine Boston Massachusetts USA; ^8^ Whitaker Cardiovascular Institute Boston University Chobanian & Avedisian School of Medicine Boston Massachusetts USA; ^9^ The University of Texas School of Public Health San Antonio San Antonio Texas USA; ^10^ The University of Texas Health Science Center San Antonio Texas USA; ^11^ Cardiovascular Engineering, Inc. Needham Massachusetts USA

**Keywords:** aortic spring, diastolic function, left ventricular‐arterial coupling, mechanical load, vascular hemodynamics

## Abstract

Mechanical twisting and untwisting of the left ventricle as well as abnormal hemodynamic aortic‐ventricular coupling are recognized as contributors to left ventricular diastolic function. However, energy associated with proximal aortic stretch during systole can be recovered as diastolic elastic recoil that may facilitate diastolic left ventricular filling. To investigate this “aortic spring” mechanism, we assessed the cross‐sectional and longitudinal associations of systolic atrioventricular plane displacement (AVPD), a surrogate measure of stretch of the mechanically coupled ascending aorta, with measures of left ventricular diastolic function. At two examinations (14 ± 1 years apart) in Framingham Heart Study participants (*N* = 7117; mean age 50 years, 55% women), we assessed AVPD and left ventricular diastolic function using echocardiography. In cross‐sectional analyses, higher AVPD was associated with higher e' (*β* per SD ± standard error = 0.49 ± 0.01; *p* < 0.001) and lower E/e' (*β* = −0.39 ± 0.01; *p* < 0.001). In longitudinal models, greater change in (Δ) AVPD between visits was associated with higher Δe' (*β* = 0.38 ± 0.01; *p* < 0.001) and lower ΔE/e' (*β* = −0.19 ± 0.01; *p* < 0.001). We observed effect modification (interaction *p*‐values: <0.001 to 0.03) for cross‐sectional and longitudinal associations by median age, sex, obesity status, hypertension treatment, and the extent of aortic stiffness (assessed via carotid‐femoral pulse wave velocity). Thus, the aortic spring function may contribute to left ventricular diastolic function.

## 1 INTRODUCTION

Diastolic dysfunction of the left ventricle (LV) is characterized by impaired ventricular relaxation and elevated cardiac stiffness and ventricular filling pressures (Wan et al., 2014). Abnormal hemodynamic coupling of the LV and aorta has long been recognized as a contributor to left ventricular diastolic dysfunction (Ikonomidis et al., 2019). However, research on the role of direct mechanical coupling of the LV and aorta in left ventricular diastolic function and subsequent disease risk is more limited.

In Framingham Heart Study (FHS) participants, we reported that aortic stiffening increases mechanical load on the LV and is associated with lower left ventricular long‐axis shortening (Bell et al., 2017). Importantly, energy stored in stretch of the spring‐like ascending aorta during systole can be recovered as elastic recoil during diastole (Bell et al., 2017). During systole, the LV shortens longitudinally, displacing the atrioventricular plane toward the apex (Hoffman & Ritman, 1985; Mitchell, 2018, 2021; Rogers Jr. et al., 1991). The distal ascending aorta is relatively immobile at the level of the brachiocephalic origin and beyond, whereas the proximal ascending aorta is tethered to the highly mobile base of the heart. Systolic displacement of the atrioventricular plane pulls on the aortic root and contributes to stretch of the ascending aorta during systole (Mitchell, 2021). Therefore, atrioventricular plane displacement (AVPD) reflects both LV long axis systolic function and mechanical stretch of the proximal aorta associated with aortic spring function. Additionally, we have posited that diastolic elastic recoil of the stretched ascending aorta exerts a reciprocal force on the left ventricular long axis, contributing to early diastolic suction (Mitchell, 2018).

In a cross‐section of Age, Gene/Environment Susceptibility (AGES)‐Reykjavik participants, impairment of the aortic spring mechanism was associated with lower early left ventricular diastolic filling, particularly in women (Bell et al., 2015), potentially contributing to disproportionate heart failure with preserved ejection fraction (HFpEF) risk in older women. In addition, aging and obesity contribute to HFpEF risk and are associated with stiffening of the aorta (Ho et al., 2016; Mitchell, Wang, et al., 2010; van den Munckhof et al., 2017). Although higher aortic stiffness may not be independently related to incident HFpEF (Pandey et al., 2017; Tsao et al., 2015), it may exacerbate the effects of existing risk factors and comorbidities that modify HFpEF risk (Paulus & Tschope, 2013). Together, these findings suggest that aortic stiffening could impair mechanical coupling of the LV and proximal aorta and contribute to left ventricular systolic and diastolic dysfunction.

The associations of longitudinal changes in cardiovascular disease (CVD) risk factors and measures of aortic spring function with longitudinal progression of left ventricular diastolic dysfunction have not been examined. We hypothesize that aortic stiffening increases the systolic load on the LV, particularly along its long axis, which reduces aortic longitudinal stretch during systole, reduces the potential energy stored in the aortic spring, and thereby impairs left ventricular diastolic function. Thus, we assessed the cross‐sectional and longitudinal associations of AVPD, a surrogate measure of aortic stretch, with measures of left ventricular diastolic function in FHS participants across the full adult age spectrum.

## 2 MATERIALS AND METHODS

### 2.1 Study sample

Our study followed the Strengthening the Reporting of Observational Studies in Epidemiology (STROBE) reporting guidelines (Von Elm et al., 2007). The sample was drawn from the Framingham Offspring, New Offspring Spouse, Third Generation, and Omni‐1 and Omni‐2 cohorts, which have been described previously (Kannel et al., 1979; Quan et al., 1997; Splansky et al., 2007). Omni participants were Black, Asian, or Hispanic individuals who resided in the MetroWest area of Massachusetts. We included participants at two examination cycle visits at which arterial tonometry and echocardiography were routinely performed. At baseline (visit 1), Framingham Offspring (*N* = 3021) and Omni‐1 (*N* = 298) participants at examination cycles 8 and 3, respectively, as well as Third Generation (*N* = 4095), New Offspring Spouse (*N* = 103), and Omni‐2 (*N* = 410) participants at examination cycle 1, were candidates for this investigation. However, 107 participants were ineligible due to offsite visits with limited examination. Of the 7820 eligible participants, we excluded participants who were missing data for independent or dependent variables (*N* = 161) and covariate data (*N* = 542) for the primary analyses. At visit 2, Framingham Offspring (*N* = 1501) and Omni‐1 (*N* = 197) participants at examination 10 as well as Third Generation (*N* = 3171), New Offspring Spouse (*N* = 56), and Omni‐2 (*N* = 294) participants at examination 3 were candidates for this investigation. Due to coronavirus disease restrictions, 317 participants were ineligible due to remote or limited examinations. Of the 4902 eligible participants, we excluded participants who were missing data for independent or dependent variables (*N* = 112) and covariate data (*N* = 481) for the primary analyses. For the longitudinal analysis, we excluded the 258 participants who were not included in both visit 1 and 2 samples. To maximize sample sizes for secondary expanded models, we excluded participants missing individual measures of cardiac structure at visit 1 on an analysis‐by‐analysis basis. Our study conforms to the Declaration of Helsinki for use of human subjects. All protocols were approved by Boston University Medical Center's Institutional Review Board, and all participants provided written informed consent.

### 2.2 Assessment of left ventricular function and structure and aortic spring engagement

We assessed mitral inflow Doppler and mitral annulus tissue Doppler in an apical 4‐chamber view with participants in the left lateral decubitus position. We measured the peak early diastolic tissue velocity of the lateral mitral annulus (e') and transmitral Doppler flow velocities (E and A wave peak velocities). We calculated E/e' as the ratio of peak early mitral inflow velocity and peak early diastolic mitral annular tissue velocity. We estimated left ventricular mass using the Devereux formula (Devereux et al., 1986) and measured diastolic left ventricular diameter and posterior and anteroseptal wall thickness using a leading‐edge technique. We calculated the left ventricular h/R ratio as the ratio of the wall thickness (i.e., the sum of left ventricular posterior wall and the left ventricular septum) divided by chamber diameter. We indexed left ventricular mass to body surface area (using the Du Bois formula).

We assessed AVPD using tissue Doppler echocardiography. We measured the displacement of the base of the heart by using the integral of the tissue Doppler s' wave. The s' wave represents the time‐resolved LV longitudinal shortening velocity during systole (Seo et al., 2010). Integration of this velocity waveform yields the total AVPD, which corresponds to the movement of the atrioventricular plane toward the apex of the heart during systole. Since the aortic arch remains stationary during the cardiac cycle (Hoffman & Ritman, 1985; Mitchell, 2018, 2021; Rogers Jr. et al., 1991), AVPD will be associated with longitudinal stretch of the ascending aorta (Bell et al., 2014, 2015). Thus, this technique provides an estimate of the change in length of the ascending aorta during the cardiac cycle. In Framingham Offspring and Third Generation participants, we measured mitral annular peak systolic excursion in the apical four‐chamber view (Hu et al., 2013). We measured systolic excursion of the mitral annulus from its point of greatest separation from the apex at the end of diastole to its point of closest proximity to the apex at the time of aortic valve closure using the integral of lateral mitral annulus tissue Doppler velocity, consistent with standard tissue Doppler acquisition protocols. Because septal and lateral motion of the mitral annulus may differ and the septal annulus is more directly coupled to aortic root motion, we performed an additional calibration analysis in a subset of FHS participants (Exam 10) with paired lateral and septal AVPD measurements available. In this subset, septal AVPD was modeled as a function of lateral AVPD, with age included as a modifier of the septal‐lateral relation; sex and cohort were evaluated but were not significant and were excluded. Regression coefficients from this analysis were then used to derive a septal‐equivalent AVPD for participants. This regression‐adjusted AVPD was used in downstream physiological calculations.

LV longitudinal pressure forces were estimated from the product of LV pressure and LV endocardial short‐axis area (i.e., Force=Pressure×Area). LV endocardial short‐axis area at end systole (*A*_LVES_) was calculated as $\pi {\left(\frac{\text{LVSD}}{2}\right)}^{2}$, where LVSD represents the LV diameter at end systole. LV longitudinal pressure force was then calculated as $1333.224\times P\times \frac{A_{\text{LVES}}}{1000}$, where *P* represents the cardiac pressure in mm Hg. Pressure values in mm Hg were converted to dynes/cm^2^ using the constant 1333.224, and resulting force values were converted to kdynes by division by 1000. We calculated LV end‐systolic pressure force using end‐systolic pressure, LV early‐diastolic pressure force using left atrial pressure estimated from E/e', and LV early‐diastolic pressure forces assuming left atrial pressures of 10 and 15 mm Hg. Aortic restoring force was estimated as the product of the effective aortic wall stiffness‐thickness product (*Eh*), ascending aortic longitudinal strain, and ascending aortic midwall circumference. Force ratios were calculated as the ratio of aortic restoring force to each LV longitudinal pressure‐derived force measure.

### 2.3 Comprehensive hemodynamic assessment

We performed a comprehensive hemodynamic assessment of the heart and aorta using arterial tonometry, echocardiography, and electrocardiography. We obtained arterial tonometry with simultaneous electrocardiography of the brachial, radial, femoral, and carotid arteries in supine participants using a custom tonometer as previously described (Mitchell, Hwang, et al., 2010). Next, all participants underwent echocardiographic evaluations, which included M‐mode, 2‐dimensional, pulsed‐wave Doppler, and tissue Doppler imaging (Philips Healthcare, Andover, MA) (Nayor et al., 2018). We also obtained 2‐dimensional echocardiographic images of the left ventricular outflow tract from a parasternal long‐axis view followed by pulsed Doppler of the left ventricular outflow tract from an apical 5‐chamber view. We digitized and transferred echocardiographic, tonometric, and electrocardiographic data to a core laboratory (Cardiovascular Engineering, Inc., Needham, MA) for blinded analyses. We signal‐averaged and synchronized tonometry waveforms using the electrocardiographic R‐wave. We calibrated the signal‐averaged brachial pressure waveform peak and trough to the cuff systolic and diastolic pressures, respectively. We calculated mean arterial pressure as the integral of the calibrated brachial pressure waveform (Mitchell, Hwang, et al., 2010). All other tonometry waveforms were calibrated by using brachial mean and diastolic pressures. Aortic stiffness was assessed as carotid‐femoral pulse wave velocity (CFPWV). We calculated CFPWV from tonometry waveforms and body surface measurements that adjusted for parallel transmission in the aortic arch and brachiocephalic artery (Mitchell et al., 2004).

### 2.4 Clinical evaluation and covariates

We selected CVD risk factors (as covariates and potential confounders) a priori based on literature review of reported associations with measures of aortic stiffness or left ventricular diastolic dysfunction and included height, body mass index, total/high‐density lipoprotein cholesterol ratio, triglycerides, fasting glucose, heart rate, mean arterial pressure, hypertension treatment, hyperlipidemia treatment, diabetes treatment, current smoking status, and prevalent CVD. Medical history was assessed routinely at each research examination. Age, sex, smoking status, presence of CVD, and use of antihypertensive, hyperlipidemia, and diabetes medications were assessed through questionnaires. Height (meters) and mass (kilograms) were assessed during the examination. Body mass index was calculated as mass in kilograms divided by height in meters squared. Mean arterial blood pressure and heart rate were assessed during tonometry. Serum blood glucose, triglycerides, and cholesterol levels were measured from fasting blood samples.

### 2.5 Statistical analyses

We tabulated the characteristics of the sample. We inverted CFPWV to limit heteroscedasticity; then we multiplied it by −1000 to convert units to ms/m and rectify the directionality of associations with aortic stiffness, that is, a higher value represents a stiffer aorta. The natural logarithm was used to transform triglycerides, total/high‐density lipoprotein cholesterol ratio, fasting blood glucose, body mass index, and E/e’ variables to normalize skewed distributions. Continuous measures were standardized (mean = 0, standard deviation = 1) for modeling in the primary analysis.

Cross‐sectional and longitudinal relations of AVPD, e', and E/e' with various CVD risk factors were assessed by using multivariable linear regression. Backward selection was used to identify a parsimonious set of CVD risk factors from among a panel that were separately related to AVPD, e', and E/e'. We selected CVD risk factors (as covariates and potential confounders) a priori based on literature review (Supplemental Appendix). Cross‐sectional models initially adjusted for age, age^2^, sex, and cohort. CVD risk factors were added to the model as a group for backward selection. In longitudinal models between examinations, linear regression analysis with backward elimination was used to assess associations of change in AVPD, e', and E/e' with changes in a parsimonious set of CVD risk factors. Longitudinal models were adjusted for age, age^2^, sex, cohort, and corresponding baseline AVPD or left ventricular diastolic function measure and baseline CVD risk factor values. The threshold for inclusion and removal from the models was two‐sided *p* < 0.05.

The foregoing final CVD risk factor models were used to assess cross‐sectional and longitudinal relations of AVPD with measures of diastolic function (e' and E/e'). Expanded cross‐sectional models were further adjusted for peak s' velocity (expanded model 1) and then left ventricular mass (indexed to body surface area) and left ventricular h/R ratio at visit 1 (expanded model 2). We conducted exploratory analyses to evaluate whether left atrial systolic or diastolic dimensions influenced associations between AVPD and diastolic measures as potential confounders, using age‐, sex‐, and cohort‐adjusted partial correlations (in native units). We also estimated Pearson partial correlations (in native units) to assess associations among mitral annular plane systolic excursion, AVPD, e', and E/e'; these models were adjusted for age, age^2^, sex, and cohort. To provide quantitative context for the relative magnitude of longitudinal forces acting on the left ventricle, we estimated end‐systolic and early‐diastolic LV pressure‐derived meridional forces and compared them with estimated aortic restoring forces derived from ascending aortic strain and elastic modulus. For longitudinal models of change in e’ and E/e’, we adjusted for a base model with CVD risk factors, followed by sequential addition of changes in s' and AVPD and their corresponding baseline values at visit 1. For both cross‐sectional and longitudinal models, we assessed potential effect modification (interaction) by incorporating corresponding interaction terms in the regression models. These included terms for age (above/below median), sex, presence of obesity (body mass index <30 vs. ≥30 kg/m^2^), hypertension treatment, and extent of aortic stiffness (above/below median CFPWV) at visit 1. For significant interactions, we performed stratified analyses. All statistical analyses were performed with SAS version 9.4 for Windows (SAS Institute, Cary, NC), and we considered two‐sided *p* < 0.05 as statistically significant.

## 3 RESULTS

**FIGURE 1:**
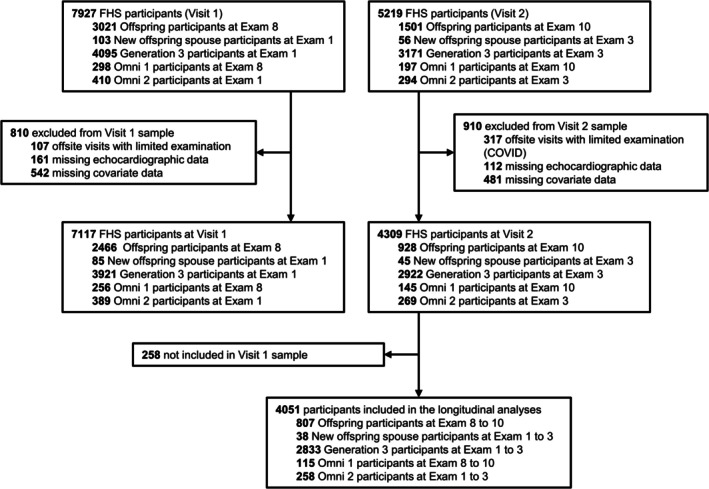
Flow diagram of the selection of the study samples. Several excluded participants engaged in offsite visits with limited examination, especially during the pandemic caused by coronavirus disease 2019 (COVID) at visit 2.

A flow chart for exclusion of participants is presented in the Figure 1 and clinical characteristics of included participants at visits 1 and 2 are presented in Table S1. The sample at visit 1 comprised relatively healthy middle‐aged and older adults, with a preponderance of Generation 3 participants, particularly at visit 2. At visit 2 as compared to visit 1, prevalences of hypertension, hyperlipidemia, and diabetes treatment were higher whereas smoking was less frequent. CFPWV and E/e' were higher, whereas e’ and s' were lower at visit 2.

Cross‐sectional associations of AVPD (s' area), s' and e' peak velocities, and E/e' with CVD risk factors at visit 1 are presented in Table S2. In models adjusted for age, age^2^, sex, and cohort, e' tended to be negatively associated and E/e' tended to be positively associated with CVD risk factors, consistent with worse diastolic function in the presence of higher CVD risk factor burden. AVPD and s' peak velocity were generally lower with higher CVD risk factor burden although higher BMI and height were associated with higher AVPD while higher heart rate, height, and presence of hypertension treatment were associated with higher s' peak velocity.

Longitudinal associations of change in (Δ) measures of AVPD and diastolic function with CVD risk factors are presented in Tables S3–S5. In general, an increase in risk factor levels was associated with a reduction in AVPD and e’ and an increase in E/e'. Interestingly, similar to cross‐sectional relations (Table S2), we observed opposing associations for change in body mass index, which was related negatively to change in e' and positively to change in AVPD and E/e'. The longitudinal CVD risk factor models explained 29%, 64%, and 51% of the variability in AVPD, Δe’, and ΔE/e', respectively.

**FIGURE 2:**
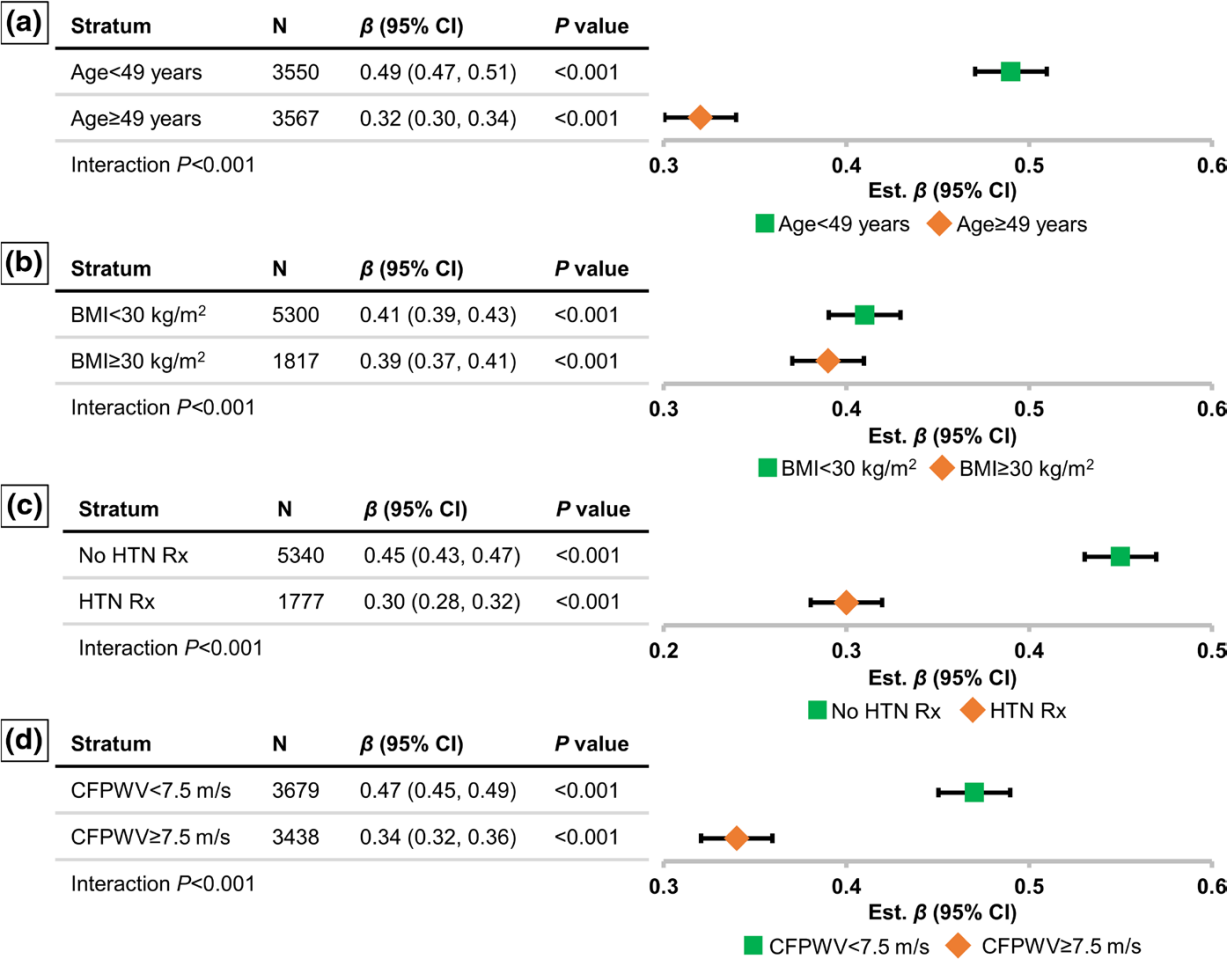
Effect modification for cross‐sectional association of atrioventricular plane displacement (AVPD) with e' by (a) median age, (b) obesity status, (c) presence of hypertension treatment, and (d) extent of aortic stiffness. Effect sizes (*β*s) and 95% CIs from stratified linear regression models. BMI, body mass index. HTN Rx, hypertension treatment. CFPWV, carotid‐femoral pulse wave velocity. Age and CFPWV groups were defined as below vs. at/above median at visit 1. Models adjust for age, age^2^, sex, cohort, height, heart rate, mean arterial pressure, body mass index, total/high‐density lipoprotein cholesterol ratio, triglycerides, prevalent hyperlipidemia treatment, and prevalent cardiovascular disease.

Cross‐sectional associations of aortic spring measures with left ventricular diastolic function are presented in Table 1. Higher AVPD was associated with higher e' (*β* per SD ± standard error = 0.49 ± 0.01; *p* < 0.001) and lower E/e' (*β* = −0.39 ± 0.01; *p* < 0.001). These associations persisted after further adjustment for differences in left ventricular contractility and structure. In addition, adjustment of partial correlations for left atrial size and s' did not meaningfully alter the associations between AVPD and diastolic measures (Table S6). Interrelations of mitral annular plane systolic excursion, AVPD, e', and E/e' are presented in Table S7. We observed a strong positive association between AVPD and mitral annular plane systolic excursion (partial *r* = 0.58; *p* < 0.0001). At mitral valve opening, the LV pressure‐derived longitudinal force decreased markedly relative to end systole, whereas the estimated aortic restoring force remained unchanged and exceeded the LV pressure‐derived force by more than an order of magnitude (Table S8). This temporal difference highlights the potential relevance of aortic restoring forces during very early diastole. Associations among AVPD, e', and E/e' are complementary to the regression analyses. Interactions for cross‐sectional relations of aortic spring measures with left ventricular diastolic function are summarized in Table S9. The association of higher AVPD with higher e' was stronger among younger participants, participants without obesity, participants without prevalent hypertension treatment, and participants with lower aortic stiffness (Figure 2). Additionally, the association of higher AVPD with lower E/e' was stronger among women (*β* = −0.36 ± 0.01; *p* < 0.001 vs. *β* = −0.30 ± 0.02; *p* < 0.001; Interaction *p* = 0.005).

**TABLE 1:** Cross‐sectional relations of atrioventricular plane displacement and left ventricular diastolic function measures at visit 1.

AVPD models	e'		E/e'	
	Est. *β* ± SE (*p*)	*R* ^2^	Est. *β* ± SE (*p*)	*R* ^2^
Base model^a^ (*N* = 7117)	0.49 ± 0.01 (<0.001)	0.69	−0.39 ± 0.01 (<0.001)	0.42
Expanded model 1^b^ (*N* = 7117)	0.51 ± 0.01 (<0.001)	0.69	−0.30 ± 0.01 (<0.001)	0.43
Expanded model 2^c^ (*N* = 6679)	0.51 ± 0.01 (<0.001)	0.69	−0.30 ± 0.01 (<0.001)	0.44

*Note*: Regression estimates (*β* ± SE) and *p* values (in parentheses) are 1 standard deviation difference in aortic stretch per 1 standard deviation difference in diastolic function measures. E/e' was natural log transformed.

Abbreviations: AVPD, atrioventricular plane displacement; e', peak early diastolic tissue velocity of the lateral mitral annulus; and E/e', ratio of peak early mitral inflow velocity and e'.

^a^ Base models for e' are adjusted for age, age^2^, sex, cohort, height, heart rate, mean arterial pressure, body mass index, total/high‐density lipoprotein cholesterol ratio, prevalent CVD, triglycerides, and hyperlipidemia treatment; base models for E/e' are adjusted for age, age^2^, sex, cohort, height, heart rate, mean arterial pressure, body mass index, total/high‐density lipoprotein cholesterol ratio, prevalent CVD, diabetes treatment, hyperlipidemia treatment, and hypertension treatment.

^b^ Expanded model 1 further adjusts for s' (peak myocardial longitudinal velocity during systole).

^c^ Expanded model 2 further adjusts for left ventricular (LV) mass (indexed to body surface area) and LV h/R ratio (the ratio of the wall thickness [i.e., the sum of LV posterior wall and the LV septum] divided by chamber diameter).

**FIGURE 3:**
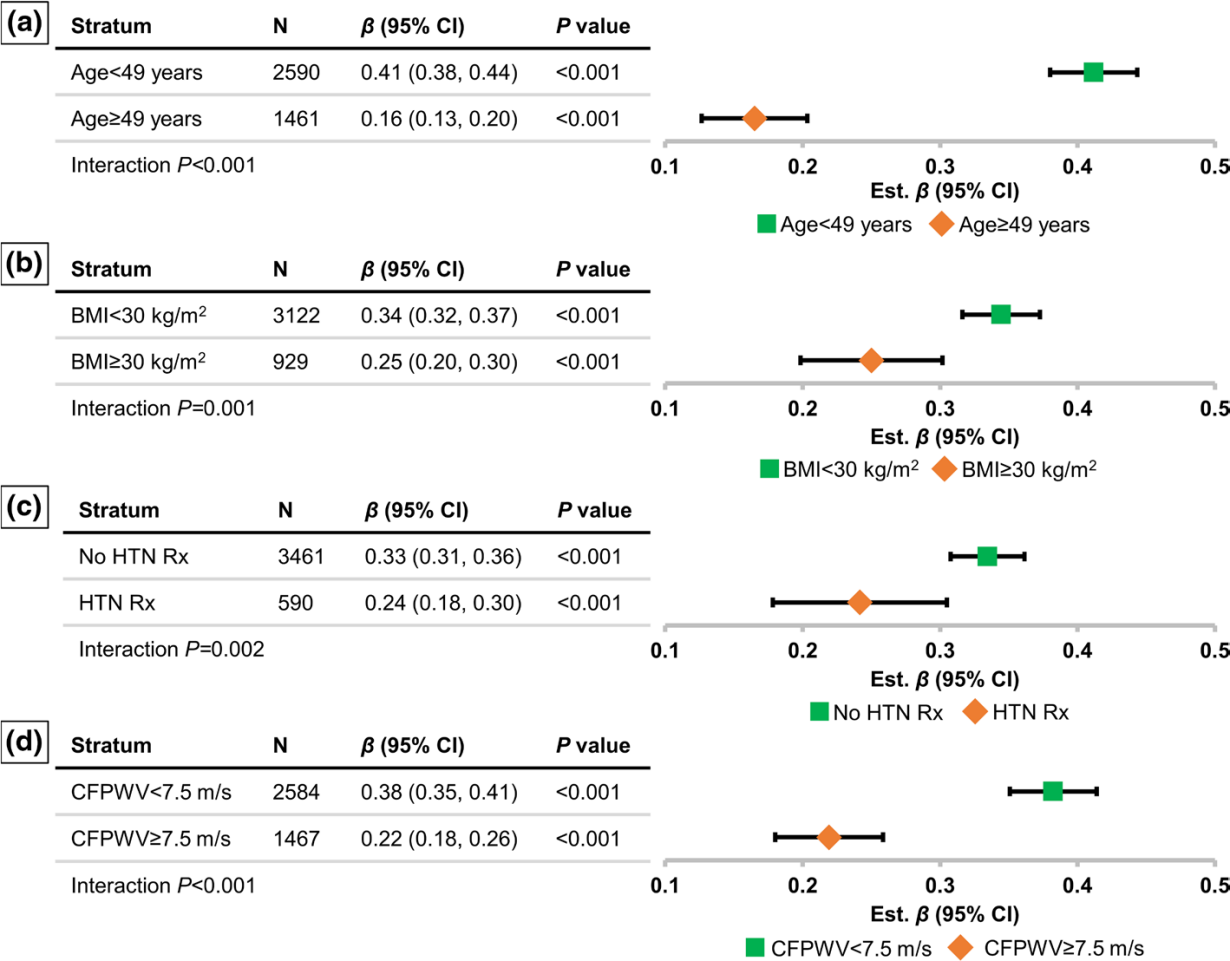
Effect modification for longitudinal associations of ΔAVPD with Δe' by (a) median age, (b) obesity status, (c) presence of hypertension treatment, and (d) extent of aortic stiffness. AVPD, atrioventricular plane displacement. Effect sizes (*β*s) and 95% CIs from stratified linear regression models. Even when the separate‐groups CIs overlap, the CI for differences (interaction) can exclude 0. HTN Rx, hypertension treatment. CFPWV, carotid‐femoral pulse wave velocity. Age and CFPWV groups were defined as below vs. at/above median at visit 1. Models adjust for age, age^2^, sex, cohort, height, baseline e' at visit 1, and baseline and corresponding longitudinal change in cardiovascular disease risk factors (heart rate, mean arterial pressure, body mass index, triglycerides, and smoking status).

**FIGURE 4:**
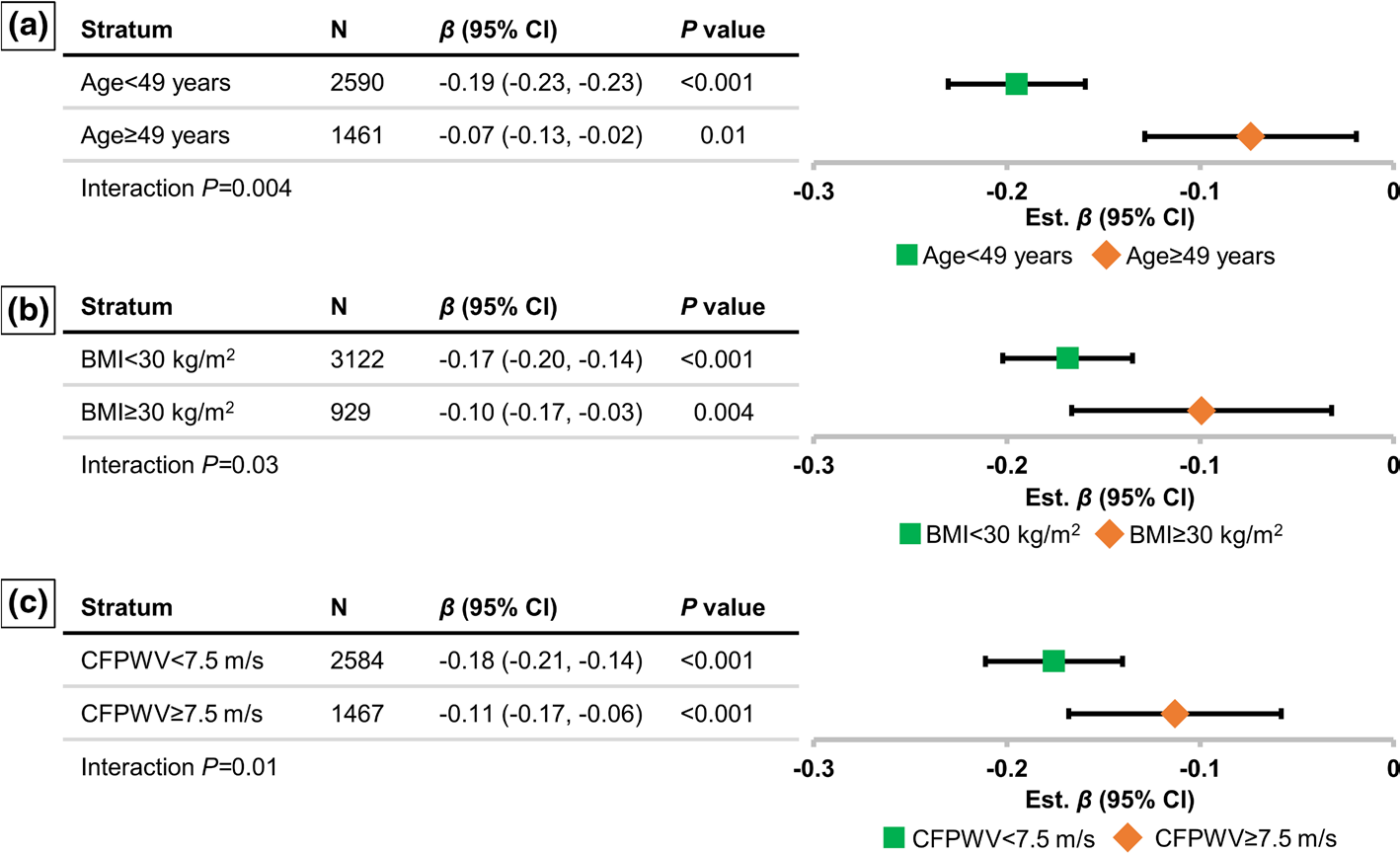
Effect modification for longitudinal associations of ΔAVPD with ΔE/e' by (a) age, (b) obesity status, and (c) extent of aortic stiffness. AVPD, atrioventricular plane displacement. Effect sizes (*β*s) and 95% CIs from stratified linear regression models. Even when the separate‐groups CIs overlap, the CI for differences (interaction) can exclude 0. CFPWV, carotid‐femoral pulse wave velocity. Age and CFPWV groups were defined as below vs. at/above median at visit 1. Models adjust for age, age^2^, sex, cohort, height, baseline e' at visit 1, and baseline and corresponding longitudinal change in cardiovascular disease risk factors (heart rate, mean arterial pressure, body mass index, fasting glucose, and smoking status).

Associations of longitudinal change in aortic spring measures with change in left ventricular diastolic function between visits are presented in Table 2. An increase in AVPD was associated with an increase in e' (*β* = 0.38 ± 0.01; *p* < 0.001) and a reduction in E/e' (*β* = −0.19 ± 0.01; *p* < 0.001). These associations persisted after further adjustment for differences in left ventricular contractility and structure. A summary of interactions for longitudinal relations of aortic spring measures with left ventricular diastolic function is presented in Table S10. The association of an increase in AVPD with an increase in e' was stronger among younger participants, participants without obesity, participants without prevalent hypertension treatment, and participants with lower aortic stiffness (Figure 3). Additionally, the association of an increase in AVPD with a reduction in E/e' was stronger in younger participants, participants without obesity, and participants with lower aortic stiffness (Figure 4).

**TABLE 2:** Relations of longitudinal changes in atrioventricular plane displacement with longitudinal changes in left ventricular diastolic function measures.

Model	AVPD variables	Δe'			ΔE/e'		
		Est. *β* ± SE (*p*)	*p* for paired AVPD variables	*R* ^*2*^	Est. *β* ± SE (*p*)	*p* for paired AVPD variables	*R* ^*2*^
Base model^a^ (*N* = 4051)	‐‐	‐‐	‐‐	0.45	‐‐	‐‐	0.32
AVPD model^b^ (*N* = 4051)	Baseline AVPD	0.12 ± 0.02 (<0.001)	<0.001	0.55	−0.07 ± 0.02 (<0.001)	<0.001	0.35
	ΔAVPD	0.38 ± 0.01 (<0.001)			−0.19 ± 0.02 (<0.001)		
Expanded model 1^c^ (*N* = 4051)	Baseline AVPD	0.08 ± 0.02 (<0.001)	<0.001	0.55	−0.01 ± 0.02 (0.56)	<0.001	0.35
	ΔAVPD	0.36 ± 0.01 (<0.001)			−0.16 ± 0.02 (<0.001)		
Expanded model 2^d^ (*N* = 3886)	Baseline AVPD	0.08 ± 0.02 (<0.001)	<0.001	0.55	−0.01 ± 0.02 (0.76)	<0.001	0.35
	ΔAVPD	0.36 ± 0.01 (<0.001)			−0.16 ± 0.02 (<0.001)		

*Note*: ΔE/e' was natural log transformed. Regression estimates (*β* ± SE) and *p* values (in parentheses) are 1 standard deviation change in aortic stretch per 1 standard deviation change in diastolic function measures.

Abbreviations: AVPD, atrioventricular plane displacement; e', peak early diastolic tissue velocity of the lateral mitral annulus; E/e', ratio of peak early mitral inflow velocity and e'; Δs', peak myocardial longitudinal velocity during systole.

^a^ Base models include age, age^2^, sex, cohort, height, baseline diastolic function measure (either e' or E/e' at visit 1), and baseline and corresponding longitudinal change in cardiovascular disease risk factors (for Δe': heart rate, mean arterial pressure, body mass index, triglycerides, and smoking status; and for ΔE/e': heart rate, mean arterial pressure, body mass index, fasting glucose, and smoking status).

^b^ AVPD model includes all covariates in the base models as well as ΔAVPD with its corresponding baseline values at visit 1.

^c^ Expanded model 1 includes all covariates in the base model as well as ΔAVPD and Δs' (peak myocardial longitudinal velocity during systole) with their corresponding baseline values at visit 1.

^d^ Expanded model 2 further adjusts for baseline left ventricular (LV) mass (indexed to body surface area) and LV h/R ratio (the ratio of the wall thickness [i.e., the sum of LV posterior wall and the LV septum] divided by chamber diameter). The secondary expanded models include additional covariates, but the number of complete observations decreased due to missing data on the added predictors.

## 4 DISCUSSION

We investigated the cross‐sectional and longitudinal relations of AVPD, a surrogate of stretch of the ascending aorta and aortic spring function, with measures of left ventricular diastolic function across a broad age range in participants in the community‐based FHS. In cross‐sectional analyses, higher AVPD was associated with better left ventricular diastolic function (higher e' and lower E/e') in models that adjusted for CVD risk factors. Relations persisted in models that further adjusted for left ventricular contractility (s' velocity) (Seo et al., 2010) as well as measures of left ventricular mass and concentric remodeling. For e', we observed evidence of effect modification by age, obesity status, presence of hypertension treatment, and extent of aortic stiffness, and for E/e', we observed evidence of effect modification by sex. In longitudinal analyses, an increase in AVPD between examinations was associated with an increase in e' and a reduction in E/e', meaning that a reduction in AVPD between visits was associated with worsening left ventricular diastolic function. The longitudinal association of an increase in AVPD with an increase in e' was stronger in younger participants, participants without obesity, participants without hypertension treatment, and participants with lower aortic stiffness. Additionally, the longitudinal association of an increase in AVPD with a decrease in E/e' was stronger among younger participants, participants without obesity, and participants with lower aortic stiffness. Thus, our results are consistent with the hypothesis that AVPD, a surrogate for stretch of the ascending aorta during systole and aortic spring function, plays an important role in maintaining left ventricular diastolic function, with putative effects modified by the extent of aortic stiffness, obesity status, age, and sex.

Multiple studies suggest that a majority (~60%–80%) of left ventricular stroke volume and much of early diastolic filling is attributable to AVPD (Carlsson, Ugander, Heiberg, & Arheden, 2007; Carlsson, Ugander, Mosen, et al., 2007; Emilsson et al., 2001; Ugander et al., 2010), in part due to fixed total heart volume because of pericardial constraint (Bowman & Kovacs, 2003; Hoffman & Ritman, 1985). Apical AVPD during systole acts like the piston in a cylinder, ejecting blood out from the LV while simultaneously pulling blood into the atria (Bell & Mitchell, 2015). Because the total (external) volume of the heart is fixed and myocardium is incompressible, modest left ventricular global longitudinal strain (12%) produces a 5‐fold higher ejection fraction (60%); conversely, a modest decline in global longitudinal strain can substantially reduce left ventricular ejection fraction (Emilsson et al., 2001, 2006; Emilsson & Wandt, 2000). In contrast, studies focused on mitral annular dynamics suggest a more distributed model of left ventricular mechanics, attributing a smaller role (~20%) to total AVPD, emphasizing instead excursion and shape dynamics involving significant radial and rotational components (Carlhall et al., 2004; Carlhall et al., 2007). The moderate correlation between AVPD assessed as the integral of the s' wave and mitral annular peak systolic excursion (partial *r* = 0.58) suggests that while both measures capture aspects of longitudinal shortening of the LV, they are not redundant.

Left ventricular torsion and compression of the elastic protein titin and its associated proteins within the myocardial cells have been portrayed as key elements of elastic energy storage during systole that enhances filling of the ventricle during diastole (Esch & Warburton, 2009; Fukuda et al., 2001; Helmes et al., 2003). If LV ejection results in an end‐systolic volume that is less than the zero pressure volume (V_0_) of the LV, there will be a modest restorative force that produces a small amount of suction that will facilitate early filling (Hori et al., 1982). Intrinsic ventricular elastic recoil and suction, particularly when end‐systolic volume approaches or falls below V_0_, have been demonstrated experimentally and may contribute to early diastolic filling (Nikolic et al., 1988). We acknowledge alternative conceptual frameworks in which diastolic suction reflects negative dP/dV during filling and contributions from residual myocardial strain, extracellular matrix architecture, and residual stress within the LV wall (Omens & Fung, 1990; Zhang et al., 2008). Diastatic LV volume exceeding V_0_ likely reflects physiological loading conditions (e.g., left atrial and intrathoracic pressures) rather than intrinsic recoil beyond V_0_. Regardless of the precise mechanistic framework, the longitudinal forces associated with such intrinsic recoil are likely small relative to those generated by end‐systolic stretch of the ascending aorta (Table S8), consistent with prior observations (Bell et al., 2015). These mechanisms are likely complementary rather than competing contributors to early diastolic LV filling. Additionally, the left ventricular pressure‐volume relation is relatively flat in the low pressure and volume range, suggesting that the magnitude of suction generated by intrinsic recoil alone is modest (Burkhoff et al., 2005; Zile et al., 2004). Furthermore, early diastolic filling normally results in a left ventricular volume that is considerably greater than V_0_ (Dokainish, 2015; Robinson et al., 2024). Thus, left ventricular recoil, per se, may only account for a small fraction of early diastolic filling. Foundational works showed that an external and antecedent preload is necessary to initiate proper diastolic filling in isolated hearts or elongation of cardiac muscle strips (Downing & Sonnenblick, 1964; Parmley & Chuck, 1973; Patterson et al., 1914; Rutlen & Powell, 1973; Sonnenblick, 1962; Starling et al., 1965; Suga & Sagawa, 1974; Weiss et al., 1976). Thus, the intrinsic cardiac elastic properties alone (i.e., recoil of energy stored internally in titin springs as a result of a preceding systolic contraction) cannot fully account for brisk early diastolic left ventricular filling (Helmes et al., 1996; LeWinter & Granzier, 2010).

Left ventricular long axis contraction (global longitudinal strain) stretches the proximal aorta as the cardiac base descends, generating considerable aortic force comparable to that produced by the LV for pressure development (Bell et al., 2015). In our participants, greater AVPD, which implies greater aortic stretch, was associated with better left ventricular diastolic function. The observations in the current study are consistent with prior work by Karwat et al. who observed moderate positive correlations between measures of aortic elastic recoil following systolic stretch (aortic root diastolic displacement and aortic root diastolic velocity) and e' in a small sample of healthy adults (Karwat et al., 2021). In addition to aortic stretch and recoil, multiple studies demonstrate that the aorta twists and untwists between systole and diastole (Amofa et al., 2019; Berdajs et al., 2016; Kim et al., 2023; Yuan et al., 2024), which further modulates mechanical ventricular‐aortic coupling and upstream LV mechanics. Thus, we posit that the interplay of aortic axial and torsional motion along with left ventricular recoil effects due to titin‐mediated untwisting contribute to early left ventricular diastolic filling through different yet complementary mechanisms. We acknowledge that AVPD also reflects ventricular longitudinal shortening and ventricular remodeling; therefore, the observed associations likely reflect integrated ventricular‐vascular interactions rather than a purely aortic process.

The relations of AVPD with e’ were diminished in the presence of a stiffer aorta and among older participants. The force required to stretch the aorta during systole increases with higher aortic stiffness, which stores more energy but also exerts an increased mechanical load on the LV. A higher load on the LV may limit the recovery of stored aortic wall energy because of adverse effects on myocyte structure and function (Bell et al., 2015; Bell & Mitchell, 2015). Additionally, storing more energy at lower systolic displacement because of a stiffer aortic wall will necessarily result in lower restorative displacement during diastole. Alternatively, aortic elongation in relation to aortic aging and stiffening may reduce the amount of preload on the aorta at the beginning of systole and may thereby limit the amount of actual stretch of and energy stored in the proximal aorta at any given level of AVPD.

The cellular environment of the aorta may play a role in the differences we observed in relations of AVPD with e’ by age and extent of aortic stiffness. Animal models of aging show that vascular smooth muscle cells isolated from aged aortas can display a senescent phenotype that contributes to aortic stiffness (Durik et al., 2012; Qiu et al., 2010; Sehgel et al., 2015). Accumulation of hypertrophied or senescent vascular smooth muscle cells in the aorta can markedly increase wall viscosity, which can limit aortic stretch during systole. Additionally, viscous losses will increase the dissipation of some of the stored energy (due to aortic stretch) as heat rather than as recovery of elastic recoil and enhanced left ventricular diastolic function (Armentano et al., 2006).

In cross‐sectional analyses, we observed more favorable effects of aortic stretch on ΔE/e’ (reflecting lower filling pressures) in women. Notably, the sex interaction was not present in longitudinal analyses, suggesting stage‐dependent effects. Our observations contrast with findings from the AGES cohort, where the association of higher stretch‐related aortic work with greater early diastolic filling was observed only in men (Bell et al., 2015). Compared to the present study, however, AGES participants were older and comprised a narrower age range, and women in AGES had lower early ventricular filling (assessed by early diastolic left ventricular filling volume on cardiac magnetic resonance imaging). Together, these findings suggest that while studies in older populations may reflect diminished early diastolic filling in women, in midlife women may exhibit a stronger coupling between aortic mechanics and left ventricular filling pressures. This may indicate a greater reliance on the aortic spring function to support early diastolic filling. Furthermore, women are more susceptible to the development of HFpEF (Russo et al., 2012; Shim et al., 2011). Previous studies have shown that women have higher left ventricular stiffness (Redfield et al., 2005), slower left ventricular relaxation (Okura et al., 2009), and greater decline in diastolic long‐axis myocardial velocities with increasing age (Foll et al., 2010). Thus, rather than an early loss of aortic spring function, aging‐related changes in ventricular structure and function may reduce the ability to translate aortic elastic energy into favorable filling dynamics, potentially contributing to the higher propensity for HFpEF in women. Further studies investigating vascular mechanisms underlying sex differences in HFpEF are needed.

## 5 LIMITATIONS

Our study has limitations that should be considered. Although we used a prospective design with cross‐sectional and longitudinal models, all associations of aortic spring measures with measures of left ventricular diastolic function were observational and cannot establish causality. We cannot dismiss the possibility of residual confounding by unknown or unmeasured risk factors (including differences in biochemical properties of titin or left ventricular collagen deposition). Because septal mitral annulus tissue Doppler was not uniformly available, AVPD was derived from lateral annular motion and calibrated using paired septal‐lateral measurements in an older FHS subset. This regression‐based approach assumes stability of the septal‐lateral relation across exams and may smooth individual differences, potentially underestimating septal displacement. Across exam visits, AVPD increased slightly while diastolic function worsened (e’ decrease while E/e’ increased). From these data, one may infer that the extent to which AVPD represents aortic stretch (and subsequent recoil capacity) may be overestimated as the ascending aorta elongates and stiffens with advancing age. In the extreme case, this lengthening of a stiffened aorta to the point that there is compression rather than persistent stretch of the aorta in late diastole could impede rather than facilitate filling. Importantly, the direction of the primary association is consistent in cross‐sectional and longitudinal analyses.

We acknowledge that ceiling effects could have contributed to observed interactions. However, the interactions between strata and effects within strata are strong and highly significant, so ceiling effects are unlikely the primary explanation. Additionally, we observed significant interaction by hypertension treatment for the associations of AVPD with e’ and E/e’. Given that we did not adjust for specific antihypertensive medications as well as the observational nature of our study, we cannot interpret these observations as a drug effect. However, our data suggest that more severe hypertension (and corresponding higher probability of hypertension treatment) may affect the relations. We acknowledge that gender norms (and associated health behaviors) may contribute to observed differences in associations by sex. We did not measure aortic strain directly because it cannot be assessed using echocardiography, and direct assessment of aortic strain using magnetic resonance imaging was not feasible in this large sample. Similarly, a fully analogous estimate of LV myocardial restoring force relative to aortic restoring force would require direct assessment of myocardial stiffness and longitudinal deformation, which were not available in the present study. Therefore, we compared aortic restoring force with pressure‐derived longitudinal chamber force and additionally adjusted expanded models for LV mass index and LV h/R ratio to partially account for LV myocardial structure and remodeling. Our study is susceptible to type‐1 error since we did not adjust for multiple testing. Yet, we observed strong associations in the primary and secondary analyses to assess effect modification that would survive multiple testing adjustments. Our samples were mostly composed of White individuals of European ancestry; therefore, our findings may not be generalizable to other ethnic or racial groups.

## 6 CONCLUSION

In FHS participants, we observed that higher AVPD, implying greater aortic spring engagement (greater longitudinal stretch of the ascending aorta), was related to better left ventricular diastolic function. Importantly, relations of greater AVPD with better early diastolic left ventricular filling (e’ peak) and lower filling pressure (E/e’) were independent of measures of contractility (s' peak). Our observations support the hypothesis that mechanical coupling between the LV and ascending aorta contributes importantly to early diastolic elongation and filling of the LV. Impairment of aortic spring function due to stiffening or elongation of the aorta or reduced AVPD due to reduced longitudinal shortening of the LV during systole, may contribute to the progression of left ventricular diastolic dysfunction. Moreover, elevated aortic stiffness, obesity, aging attenuate the aortic spring mechanism over time, which may make these groups more susceptible to left ventricular diastolic dysfunction and subsequent heart failure, particularly HFpEF. Further research on the mechanical and hemodynamic mechanisms that precede diastolic dysfunction is warranted to inform better therapeutic and preventative strategies.

## AUTHOR CONTRIBUTIONS

**Leroy L. Cooper:** Conceptualization; funding acquisition; investigation; methodology; project administration; supervision; visualization. **Brenton R. Prescott:** Formal analysis. **Vanessa Xanthakis:** Supervision. **Jian Rong:** Formal analysis. **Martin G. Larson:** Supervision. **Emelia J. Benjamin:** Funding acquisition. **Naomi M. Hamburg:** Funding acquisition. **Ramachandran S. Vasan:** Funding acquisition. **Gary F. Mitchell:** Conceptualization; funding acquisition; investigation; methodology; project administration.

## FUNDING INFORMATION

This work was supported by the National Heart, Lung, and Blood Institute through contracts [N01‐HC‐25195, HHSN268201500001I, 75N92019D00031 (Vasan)] and grants [HL071039, HL077447, HL080124, HL093328, HL104184, HL107385, HL126136, HL142983, AG079390, HL131532, HL143227 (Vasan, Mitchell); HL04334 (Vasan); HL073551, HL094898 (Mitchell); AG028321, HL060040, AG066010, HL070100, HL076784, HL092577, HL128914 (Benjamin); 2U54HL120163, U54HL120163 (Hamburg, Benjamin); HL115391, HL168889 (Hamburg); K01HL161494 (Cooper)]. The American Heart Association supported this work through grants 18SFRN34110082 (Benjamin) and 20SRFRN35120118 (Hamburg). The National Institute of Diabetes and Digestive and Kidney Diseases supported this work through grants DK082447 (Mitchell) and DK080739 (Vasan). Dr. Vasan was supported in part by the Evans Medical Foundation and the Jay and Louis Coffman Endowment from the Department of Medicine, Georgetown University School of Medicine.

## CONFLICT OF INTEREST STATEMENT

Dr. Mitchell is owner of Cardiovascular Engineering, Inc., a company that designs and manufactures devices that measure vascular stiffness. The company uses these devices in clinical trials that evaluate the effects of diseases and interventions on vascular stiffness. G.F.M. also serves as a consultant to and receives grants and honoraria from Novartis, Merck, Bayer, Servier, Philips, and deCODE genetics. The remaining authors have no disclosures to report.

## ETHICS STATEMENT

Our study conforms to the Declaration of Helsinki for use of human subjects. All protocols were approved by Boston University Medical Center's Institutional Review Board, and all participants provided written informed consent.

## Supporting information


**Table S1.** Clinical characteristics at baseline and follow‐up.
**Table S2.** Cross‐sectional relations of atrioventricular plane displacement, s', e', and E/e' with cardiovascular disease risk factors at visit 1 (*N* = 7117).
**Table S3.** Relations of change in atrioventricular plane displacement with changes in cardiovascular disease risk factors between two visits (*N* = 4051).
**Table S4.** Relations of change in e' with change in cardiovascular disease vascular risk factors between two visits (*N* = 4051).
**Table S5.** Relations of change in E/e' with change in cardiovascular disease vascular risk factors between two visits (N = 4051).
**Table S6.** Partial correlations between diastolic function measures and AVPD with and without adjustments for left atrial size (*N* = 7101).
**Table S7.** Matrix of Pearson correlation coefficients for mitral annular plane systolic excursion, AVPD, e', and E/e' (*N* = 6387).
**Table S8**. Estimated longitudinal forces acting on the left ventricle at end systole and early diastole at visit 1 (*N* = 6607).
**Table S9.** Summary of interactions for relations of atrioventricular plane displacement with left ventricular diastolic function at visit 1.
**Table S10.** Summary of interactions for longitudinal relations of atrioventricular plane displacement with left ventricular diastolic function.

## Data Availability

The data underlying this article will be shared upon request. The procedures for requesting data from the Framingham Heart Study can be found at https://www.framinghamheartstudy.org/.
